# Cytotoxicity Mechanisms of Blue-Light-Activated Curcumin in T98G Cell Line: Inducing Apoptosis through ROS-Dependent Downregulation of MMP Pathways

**DOI:** 10.3390/ijms24043842

**Published:** 2023-02-14

**Authors:** Saad Alkahtani, Norah S. AL-Johani, Saud Alarifi, Mohd Afzal

**Affiliations:** 1 Department of Zoology, College of Science, King Saud University, P.O. Box 2455, Riyadh 11451, Saudi Arabia; 2 Department of Chemistry, College of Science, King Saud University, P.O. Box 2455, Riyadh 11451, Saudi Arabia

**Keywords:** glioblastoma, curcumin, neurodegenerative disorders, photodynamic therapy, reactive oxygen species, antioxidant

## Abstract

We examined the photodynamic activation of Curcumin under blue light in glioblastoma T98G cells. The therapeutic effect of Curcumin, in both the absence and presence of blue light, was measured by the MTT assay and apoptosis progression using flow cytometry. Fluorescence imaging was carried out to evaluate Curcumin uptake. Photodynamic activation of Curcumin (10 µM), in the presence of blue light, enhanced its cytotoxic effect, resulting in the activation of ROS-dependent apoptotic pathways in T98G cells. The gene expression studies showed the expression of matrixes metalloproteinase 2 (MMP2) and 9 (MMP9) decrease with Curcumin (10 µM) under blue light exposure, indicating possible proteolytic mechanisms. Moreover, the cytometric appearance displayed that the expressions of NF-κB and Nrf2 were found to be increased upon exposure to blue light, which revealed a significant induction of expression of nuclear factor as a result of blue-light-induced oxidative stress and cell death. These data further demonstrate that Curcumin exhibited a photodynamic effect via induction of ROS-mediated apoptosis in the presence of blue light. Our results suggest that the application of blue light enhances the therapeutic efficacy of Curcumin in glioblastoma because of the phototherapeutic effect.

## 1. Introduction

Glioblastoma, commonly known as glioblastoma multiforme (GBM), is one of the most aggressive brain tumors and it develops from astrocytes or glial precursor’s alteration [1,2,3]. GBM is a common and highly aggressive form of brain cancer that is also a high-grade (grade IV) tumor. It is characterized by a high mortality rate due to its aggressiveness with patients and for having a low probability of survival [2,4]. GBM arises due to dysregulation in cellular signaling routes consequent to mutations in LeY cell survival proteins, leading to cancer development, invasion, and disease progression [5,6]. The hyperactivation of the PI3-kinase pathway, the mutation in retinoblastoma, and p53 genes are commonly reported in GBM cases [7,8]. Despite the advances in therapeutic strategies, such as neurosurgery, radiation, and chemotherapy, for the treatment of brain tumors, either the toxic side effects or radiation- or drug-induced resistance [9,10] limit their use. Therefore, the development of novel and more targeted therapies, which target only tumor cells in a non-invasive manner leaving behind healthy unaffected cells, is warranted [11].

Photodynamic therapy (PDT) has several advantages over conventional approaches, such as fewer side effects and exact targeting by selective photoactivation of “prodrug” into the active forms at the target [12,13,14]. The cytotoxic oxygen species, created photolitically, rapidly react with surrounding cells and cause cell death [12,15]. PDT, as a non-invasive treatment, has several outstanding features, some of which include exact targeting of cells by selective illumination, a much less invasive technique than surgery, having low morbidity, and it can be repeated at the same site if required [16]. It is believed that the cytotoxic potential of photoactive molecules increases several folds when excited under blue light, as compared with red light [13,17]. The hydrophobic PSs have been shown to penetrate more deeply into the cells than hydrophilic ones due to their high lipophilicity [15,18]. As such, many synthetic dyes and natural pigments could be employed as photosensitizers for PDT because they may be less prone to adverse drug interactions [19,20].

**Figure 1:**
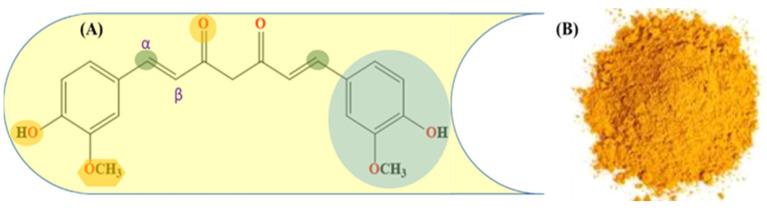
(**A**) Molecular structure of Curcumin showing the active functional group and α,β-unsaturated system; (**B**) dried and powdered rhizome of turmeric (Curcuma longa).

In recent years, the role of diet-derived herbal and natural agents has gained significant attention in the treatment of glioblastoma. Curcumin, a bioactive polyphenol (Figure 1), [15] is not only known for its antioxidant properties [21] but also possesses enormous biological features, including anti-inflammatory [22], cardioprotective [23], and neuroprotective effects [24]. Curcumin bears other physical characteristics, such as lipophilicity and a low molecular weight, that allow it to penetrate the blood–brain barrier effectively [25]. Due to this reason, it has been effectively applied as an efficient therapeutic and protective agent in neurological disorders, such as Alzheimer’s [26], Parkinson’s [27], traumatic brain injury, peripheral nerve injury, and GBM [28]. Furthermore, Curcumin-triggered apoptosis in human glioblastoma cells via mitochondria-mediated proteolytic pathways. Curcumin inhibits PI3K/Akt and NF-kB activation, downregulates Bcl-xL, and induces mitochondrial dysfunction [25,29]. More recently, Luo et al. reported that Curcumin could prevent proliferation and include apoptosis in GBM cell lines [30]. Curcumin is a light-sensitive compound and it has a wide absorption peak (300 to 500 nm), which overlaps with blue light emission [31]. It has been shown that various cancer cells show significant sensitivity to the Curcumin PDT killing effects when activated with blue LED light [32]. It has also been shown that Curcumin PDT offers therapeutic benefits for a number of ailments, including arthritis [33], cancer [34], inflammation [35], liver disease [36], metabolic syndrome [37], neurodegenerative diseases [38], obesity, and cardiovascular disease, by regulating lipid and cholesterol metabolism [39,40,41].

The study aimed to explore the photodynamic effect of Curcumin in inducing apoptosis in human malignant glioblastoma cells via a proteolytic mechanism through NF-κB and Nrf2 axes.

## 2. Results

### 2.1. Cytotoxicity and Photodynamic Implication of Curcumin

**Figure 2:**
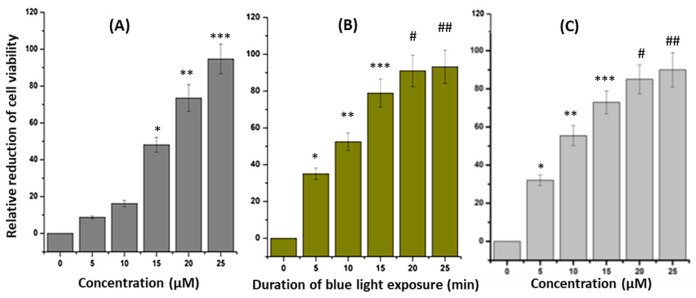
MTT assay showing: (**A**) the effect of increasing doses (0–25 µM) of Curcumin on T98G cell line after 24 h, (**B**) the effect of increasing blue light exposure (0–25 min) in T98G, and (**C**) LN229 cell line pretreated with Curcumin (10 µM). The significant results presenting *p* values < 0.05 were labeled as *. In (**A**), * denoted control versus 15 µM, ** denoted control versus 20 µM, and *** denoted control versus 25 µM. In (**B**), * denoted control versus 5 min of blue light exposure in 10 µM Curcumin pretreated T98G cells, ** denoted control versus 10 min of blue light exposure in 10 µM Curcumin-pretreated cells, and *** denoted control versus 15 min of blue light exposure in 10 µM Curcumin-pretreated cells. In the case of #, the *p* value was <0.005 (# denoted control versus 20 µM Curcumin-pretreated ## denoted control versus 25 µM Curcumin-pretreated LN229 cells).

To evaluate the photodynamic effect induced by Curcumin against the T98G cancer cell line, MTT assay was performed in the absence of blue light (Figure 2A). The T98G and LN229 cells (4 × 10^3^ cells per well) were incubated with Curcumin at increasing concentrations (0, 5, 10, 15, 20, and 25 µM). From the MTT analysis, it was observed that after 10 µM of Curcumin, there was significantly higher cell death when compared with the control untreated cells. The results also revealed a concentration-dependent increase in cell death after Curcumin treatment in both cell lines. Furthermore, 25 µM of Curcumin-treated cells exhibited greater than 90% cell death, while at 10 µM less than 20% cell death was detected. Based on this, a 10 µM Curcumin concentration was selected as the optimal dose to investigate the photodynamic effect of Curcumin under blue light (Figure 2B). In 10 µM pretreated T98G cells 43.37, 52.92, 79.32, 92.06, and 93.95% cell death was observed when blue light irradiation of 430 nm was conducted for 5, 10, 15, 20, and 25 min, respectively.

### 2.2. Confirmation of Apoptosis

**Figure 3:**
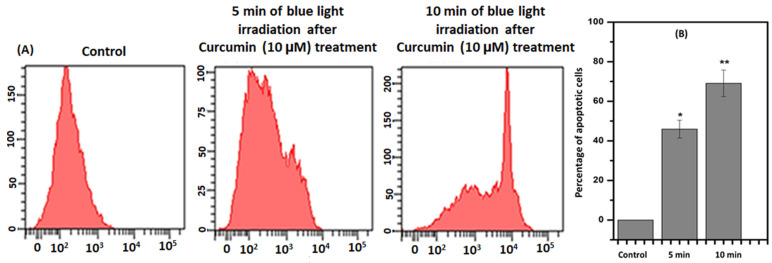
(**A**) Annexin V-FITC binding pattern of Curcumin (10 µM) treated and with the blue-light-irradiated cells at 5 and 10 min of the time interval. (**B**) The quantitative and statistical analyses of apoptotic cell death after 5 and 10 min of blue-light-irradiated to Curcumin-treated (10 µM) cells where: * denoted control versus 5 min of irradiation after Curcumin treatment (10 µM), and ** denoted control versus 20 µM. The significant results presented a *p*-value < 0.05.

To evaluate whether the photodynamic effect of Curcumin was directly correlated with apoptosis, flow cytometric analysis was utilized. It is documented that phosphatidylserine (PS), a structural component of cell membranes, is externalized during apoptosis. Annexin V binds strongly and specifically with PS that is on the extracellular surface of the cell membrane, which occurs during apoptosis (Figure 3). The 2 × 10^6^ cells were harvested in each well for Curcumin treatment along with light irradiation. After that, the cells were resuspended in the binding buffer supplied by the kit. The findings indicated that the proportion of cells positive for Annexin V-FITC staining increased as the duration of blue light increased in the presence of Curcumin. This flow cytometric experiment was performed three times to obtain the result. FlowJo software was used to find the presentable data.

### 2.3. Intracellular Reactive Oxygen Species (iROS) Generation

**Figure 4:**
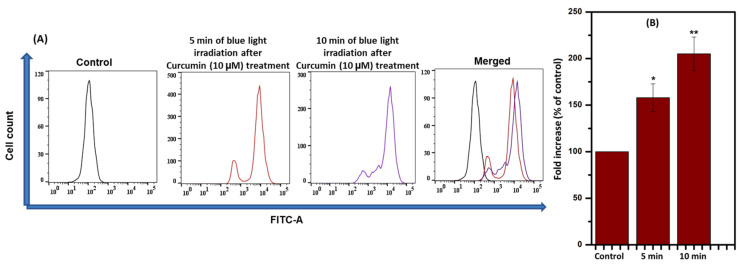
(**A**) Measurement of iROS in the presence and absence of blue light (with and without Curcumin) and (**B**) a merged image of the same. (**B**) The quantitative analysis of oxidized DCFDA levels and statistical analysis after 5 and 10 min of blue-light-irradiated 10 µM Curcumin-treated cells where: * denoted control versus 5 min of irradiation after Curcumin treatment (10 µM) and ** denoted control versus 10 min of irradiation after Curcumin treatment (10 µM). The significant results presented a *p* value < 0.05.

Apoptosis is triggered by elevated ROS, either independently or dependently. Thus, ROS was checked via flow cytometry. A cell-permeable dye, DCFH-DA, was undertaken to achieve an intracellular ROS generation experiment. T98G cells (2 × 10^6^ in each well) that were treated with Curcumin (10 µM) were studied in the absence and the presence of blue light exposure of 430 nm for 5 and 10 min. As shown in Figure 4, Curcumin-treated (10 µM) cells exhibited a moderate level of ROS generation based on the DCF fluorescence with respect to blue light exposure duration. The data confirmed that ROS production increases in the presence of blue light in Curcumin-treated T98G cells. From Figure 2, Figure 3 and Figure 4, it can be inferred that Curcumin has a probable role in photodynamic therapy in the presence of blue light via ROS generation. This flow cytometric experiment was replicated three times. FlowJo software was used to obtain the presentable data.

### 2.4. Cell Cycle Analysis

**Figure 5:**
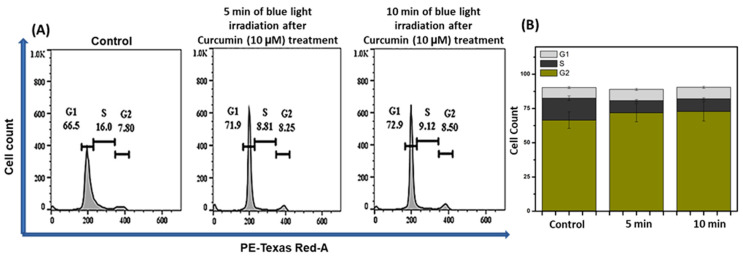
(**A**) Measurement of cell cycle arrest in the presence and absence of blue light (with and without Curcumin) and (**B**) cell death percentage in each step of the cell cycle (from **A**). (**B**) Cell death percentage in each step of the cell cycle with statistical analysis.

One of the extensively used assays to explain apoptosis through flow cytometry is the estimation of fractional DNA content [42]. The fraction of DNA content in the major phases of the cell cycle was determined to understand the PDT ability of Curcumin on cancer cells’ progression through the cell cycle [43]. In the experimental condition, 2 × 10^6^ in each well, the increase in the DNA content was observed under the influence of blue light at different time intervals applied to the Curcumin-treated (10 µM) cells. DNA content in the sub-G_0_–G_1_ phase increased to 71.9% and 72.9% after 5 and 10 min blue light exposure in Curcumin (10 µM)-pretreated T98G cells, whereas for the control, the DNA content in sub-G_0_–G_1_ is 66.5% (Figure 5). These data further confirmed that Curcumin exhibited photodynamic efficacy via ROS-mediated apoptosis in the presence of blue light of 430 nm. This flow cytometric experiment was replicated three times.

### 2.5. Expression of MMP2 and MMP9 Pathway

**Figure 6:**
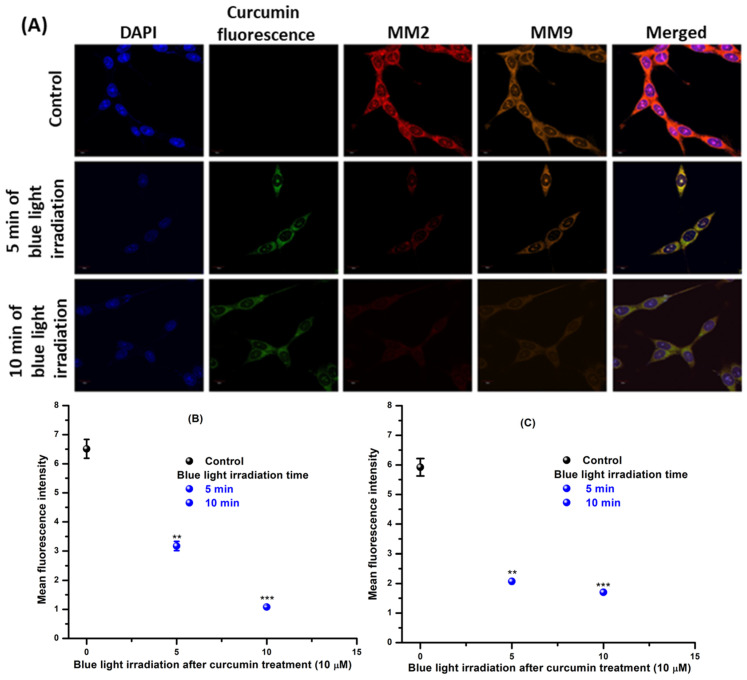
(**A**) Internalization of Curcumin (10 μM) and the expression of MMP2 and MM9 after 5 and 10 min of blue light exposure. (**B**) and (**C**) are the plots showing statistical analyses of MMP2 and MMP9, respectively. The * denoted control versus 5 min of irradiation after Curcumin treatment (10 µM), ** denoted control versus 10 min of irradiation after Curcumin treatment (10 µM) and *** denoted control versus 10 min of irradiation after Curcumin treatment (10 µM). The significant results presented a *p* value < 0.05.

Several investigations suggest that tumor growth, invasion, and metastasis are stimulated by matrix metalloproteases (MMPs), which are capable of degrading the extracellular matrix and remodeling during the course of inflammation and wound healing [44,45,46]. Therefore, increased MMP expression was noticed in cancerous tissues and was correlated with metastatic spread and unfavorable prognosis [47]. Typically, the MMP levels are low under normal conditions; however, overexpression of MMPs facilitates tumor progression, which provoked the development of MMP inhibitors (MMPIs) as cancer therapeutics [48]. In cancer cells, intracellular ROS was reported to mediate an increase in the activity of MMPs by growth factor stimulation [49]. Nevertheless, a series of signaling pathways, such as Mitogen-Activated Protein Kinase (MAPK), Phosphoinositoly-3-Kinase (PI3K), and Protein Kinase C (PKC) families, also participates in the transcriptional control of MMPs [50,51]. Therefore, the expression of MMP2 and MMP9 was evaluated for Curcumin-treated (10 µM) T98G cells after blue light irradiation (Figure 6). The fluorescence of Curcumin was also visualized under the green channel. The expression of MMP2 and MMP9 was also investigated, and Figure 6 shows that the expression of MMP2 and MMP9 decreased in a time-dependent manner to exposure to blue light irradiation of 430 nm in cells treated with Curcumin (10 µM). The statistical analysis was carried out using ImageJ (Figure 6B,C). The data confirmed that the PDT efficacy of Curcumin is due to ROS-mediated downregulation of MMPs. This flow cytometric experiment was replicated three times.

### 2.6. Expression of NF-κB and Nrf2

**Figure 7:**
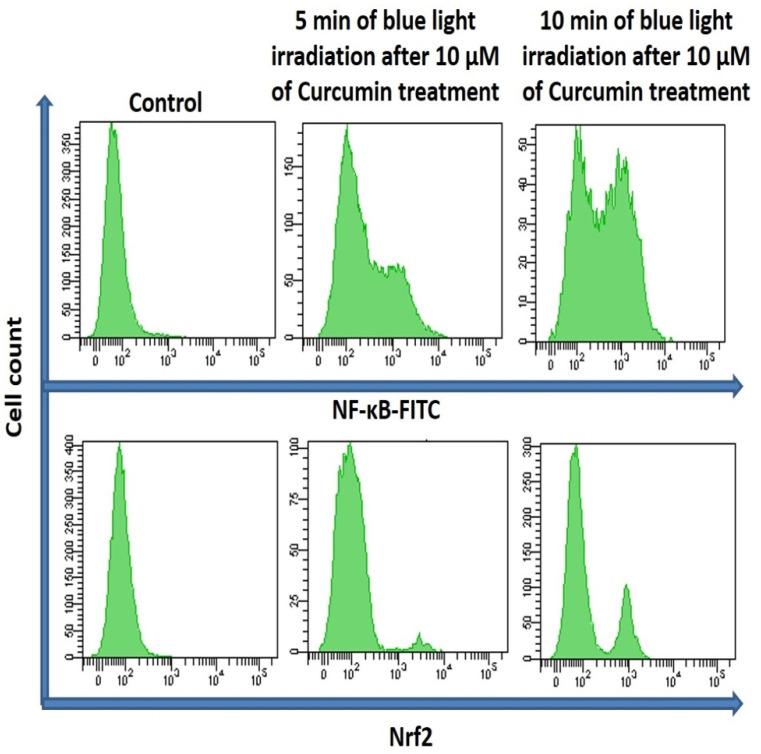
Measurement of NF-κB and Nrf2 in the presence and absence of blue light (with and without Curcumin).

Reactive oxygen species, formed by oxidative stress, are concerned with the initiation, promotion, and malignant conversion of carcinogenesis through activation/suppression of redox-sensitive transcription factors such as NF-κB and Nrf2. Therefore, the expression of NF-κB and Nrf2 was measured by flow cytometry for Curcumin-treated (10 µM) T98G cells after 5 and 10 min blue light irradiation (Figure 7). The cytometric appearance displayed that the expression of NF-κB and Nrf2 was increased in time-dependent blue light irradiation, which revealed a significant induction of expression of nuclear factor as a result of blue-light-induced oxidative stress and cell death.

## 3. Discussion

The present study evaluates the effects of irradiation time with blue light on Curcumin-mediated cell death against human glioma cells. PDT is a minimally invasive therapy to cure several cancers, based on the conversion of non-toxic molecules that are photosensitive to attack cellular structures inducing ROS after light excitation (particular wavelength) and which can cause cell death [52]. This therapy is based on the systemic application of the photosensitizer, which can target intended tissues [31]. Upon excitation of a photosensitizer at the target location by a suitable wavelength, a series of biochemical reactions are initiated, which, consequently, result in cell death. Based on this, PDT has been widely applied in the treatment of several cancers [32]. The application of PDT in oncology has considerably enhanced the anticancer potential of Curcumin [53,54,55]. The Curcumin PS can exhibit the photodynamic response via light radiation and act at a target location [56].

Curcumin was selected for this study as a natural bioactive molecule, because it has been shown to have a direct influence on endometrial invasion and adhesion, and it can induce apoptosis and angiogenesis [57], as well as activating the nuclear factor erythroid 2-related factor 2 (Nrf2)-Keap1 pathway, which is required for cell survival [58]. T98G cells were exposed to 10 µM Curcumin in the presence of blue light and we found that photoactivation enhances the cytotoxic effect of Curcumin on the cell line. In a structural study, hydroxyl groups at the ortho position on the aromatic rings and the beta-diketone in Curcumin were found to regulate phase 2 detoxification enzymes [59]. A study also found that 10 µΜ of Curcumin could lower ROS levels in rat peritoneal macrophages [60]. Furthermore, Curcumin stimulates the activity of the haemoxygenase-1 (HO-1) gene by activating the Nrf2/antioxidant response element (ARE) pathway [61]. A previous study has shown that the photoactivation of Curcumin stimulates the formation of hydrogen peroxide, which is less cytotoxic than singlet oxygen; nevertheless, its period of action is relatively longer [62]. The inflation of reactive oxygen species leads to oxidative stress, thereby directly damaging the cells. Once Curcumin enters the cell, it absorbs light of a proper wavelength, undergoes electronic transitions due to excitation, and releases electrons that can attack biological molecules [32,63,64]. The adjacent biomolecules gain energy, transfer hydrogen ions or electrons, and form free radicals and anion free radicals. Subsequently, the free radicals interact with oxygen molecules to produce ROS [65,66]. Curcumin and radiotherapy have been shown to regulate the NFκβ pathway, sensitizing colon cancer cells to chemotherapy and preventing chemoresistance [67,68]. Similarly, Curcumin treatment of a colorectal cancer cell line has been demonstrated to regulate the NFκβ pathway, suppressing the expression of key regulatory genes involved in cell survival and cell cycles, such as cyclin D1, Bcl-2, VEGF, and MMP9 [69].

In this study, the phototoxicity of Curcumin in the presence of blue light against the T98G cell line was observed. First, the cytotoxicity of Curcumin in T98G cells was checked using the MTT assay. A 10 µM concentration was selected for further measurement of its phototoxicity because, at that concentration, there was significantly less toxicity (below 20%) under blue light exposure. After that, the MTT assay was completed in Curcumin-pretreated (10 µM) blue-light-irradiated (0, 5, 10, 15, 20, and 25 min) T98G cells. The MTT data confirmed that cell death was found to be increased when the duration of blue light exposure increased. Therefore, 5 and 10 min durations for blue light irradiation, and a proper concentration of Curcumin (10 µM), were selected for further experiments.

To understand the cell death mechanism, Annexin V-FITC was used as an apoptotic marker. The flow cytometric analysis exposed that the Annexin V-FITC-positive cell population increased under blue-light-irradiated Curcumin-pretreated cells. This flow cytometry data further showed that Curcumin exhibited photodynamic implications via apoptosis in the presence of blue light. There are various reports on PDT efficacy, which have explored that it can be affected by the nature and persistence of ROS. Thus, the estimation of ROS in Curcumin-pretreated blue-light-irradiated cells was observed by DCFH-DA, and the data confirmed that ROS were increased after blue light exposure in a time-dependent manner in 10 µM Curcumin-pretreated cells. These incidents further established that the cause of the photodynamic role in T98G cells is intracellular ROS.

It has already been established that oxidative stress activates the DNA damage response (DDR) pathway and the stressed cancer cell moves toward cell cycle arrest [70]. The exhibited emission of PI confirmed that G2-M arrest results after blue light exposure in Curcumin-pretreated T98G cells. After that, the expression of MMP2 and MMP9 was measured by confocal microscopy in Curcumin-treated blue-light-irradiated T98G cells. The images confirmed that the expression of MMP2 and MMP9 was decreased in time-dependent blue light exposure after Curcumin (10 µM) treatment. The green emission of Curcumin was also visualized under a microscope. Thus, the present study concluded that Curcumin has photodynamic ability under blue light irradiation via downregulating the MMP2 and MMP9 pathways. We also checked the expression of NF-κB and Nrf2 after 10 µM Curcumin treatment in the presence of blue light exposure. The flow cytometric data established that intracellular ROS generation influences NF-κB and Nrf2.

## 4. Materials and Methods

### 4.1. Cell Lines and Chemicals

The human glioblastoma (T98G) Cellosaurus cell line (LN229) was obtained from ATCC, USA. Cell culture constituents, including fetal bovine serum (FBS), DMEM, penicillin−streptomycin−neomycin (PSN) antibiotic, EDTA, and trypsin, were obtained from Gibco (Grand Island, NY, USA). Kit for biochemical assay and inhibitors were acquired from Calbiochem (Burlington, MA, USA). Antibodies were obtained from Santa Cruz Biotechnology (Dallas, TX, USA), Abcam (Cambridge, UK), and eBioscience (San Diego, CA, USA).

### 4.2. Cell Culture and Cytotoxicity Assay

T98G and LN229 cells were cultured in DMEM, which was supplemented with 10% FBS and 1% PSN antibiotic under humidified conditions (5% CO_2_). The detailed procedure of cell culture and MTT assay adapted to the standard methods and practices [71], was also implemented in our previous work [72,73]. Here, the concentrations of Curcumin were maintained at 0–25 µM for the treatment of the glioblastoma cell line for 24 h.

### 4.3. In Vitro Photodynamic Therapy (PDT)

The photodynamic behavior of Curcumin as a photosensitizer inside the targeted cancer cells was analyzed at the optimum concentration of Curcumin (10 μM). To govern the ability of PDT, Curcumin-treated T98G cells were monitored under blue light at various time intervals (0, 5, 10, 15, 20, and 25 min) following the MTT assay.

### 4.4. Flow Cytometry

Flow cytometric analysis using Annexin V-FITC/PI staining was carried out to confirm whether photodynamic activation of Curcumin induced cell death (apoptosis/necrosis) [74]. The cells pretreated with Curcumin (10 µM) were subsequently placed under blue light for different periods of time (5 and 10 min). The cells were then washed and stained with PI and Annexin V-FITC before being analyzed in BD flow cytometer (BD LSRFortessa^TM^, San Jose, CA, USA). The number of cells 1 × 10^6^ was taken for each tube. The percentages of viable, apoptotic (early and late), and necrotic cells were recorded. This experiment was performed in triplicate.

### 4.5. Measurement of Intracellular Reactive Oxygen Species (iROS)

2,7-dichlorofluorescein diacetate (H2DCFDA) was used as an indicator of iROS generation [75]. Oxidation of H2DCFDA causes the dye to emit bright-green fluorescence at 515 nm when excited at 490 nm and, as such, can be a measure of oxidative stress induced by ROS. Here, cells were treated Curcumin (10 µM) in the absence and presence of blue light for 5 and 10 min and then incubated with H2DCFDA (30 min) in the dark. For each tube, 1 × 10^6^ cells were taken. The acquisition was achieved using a flow cytometer (BD LSRFortessa^TM^, San Jose, CA, USA). This experiment was executed in triplicate.

### 4.6. Cell Cycle Analysis

Cell cycle arrest was assayed by DNA quantification [76]. T98G cells were pretreated with Curcumin (10 µM) and placed under blue light for 5 and 10 min intervals. The cells were incubated at 4 °C overnight in ethanol (70%) followed by centrifugation. Afterward, they were resuspended into PBS possessing RNase (25 μg/mL) and incubated at 37 °C for 1 h. The cells (10^6^) were stained with PI dye (50 μg/mL) for 15 min at 4 °C. For each tube, 1 × 10^6^ cells were taken. The data were examined using FlowJo (version 10.0) software. This experiment was performed in triplicate.

### 4.7. Immunofluorescence Assay

The T98G cells were mounted onto the cover slip and incubated for 24 h taken as a control. The cells treated with Curcumin (10 μM) were irradiated as per the proposed duration. After blue light exposure, control/treated T98G cells were cleaned for 10 min in PBS and incubated for 1 h in a blocking solution. Cells were incubated with primary antibodies (MMP2 and MMP9) followed by being washed and stained with respective fluorophore-conjugated secondary antibody. The slide was counterstained for 10 min with 6-diamidino-2-phenylindole (DAPI) before being mounted with the ProLong Antifade Reagent (Molecular Probes, Eugene, OR, USA). The final image of the cells was captured using a confocal laser scanning microscope (FV10i, Olympus, Japan) [77].

### 4.8. Statistical Analysis

Data were presented as means ±SEM. Statistical significance and differences among the groups were assessed via one-way analysis of variance (ANOVA) using OriginPro 8.0 software (San Diego, CA, USA). A *p*-value ˂ 0.05 was considered significant. Post hoc analyses were conducted to indicate exactly where statistically significant differences existed.

## 5. Conclusions

To conclude, it is well recognized that glioblastoma cancers are highly invasive because of their limited treatment options. Consequently, the result showed that Curcumin combined with the PDT strategy induce ROS generation and T98G cell death via *NF-κ*B- and Nrf2-mediated MMP2 and MMP9 axis pathways in glioblastoma cancers. These results led us to realize that there is a good possibility that conventional therapies with side effects might be replaced with this dual treatment method.

## Data Availability

Not applicable.
